# Local Electrochemical Corrosion Properties of a Nano-SiO_2_/MAO Composite Coating on an AM60B-Mg Alloy

**DOI:** 10.3390/ma15113999

**Published:** 2022-06-04

**Authors:** Xiaoyu Yang, Yu Mo, Ting Dai, Jie Zhao, Yanhong Gu

**Affiliations:** 1 School of Mechanical Engineering, Beijing Institute of Petrochemical Technology, Beijing 102617, China; yangxy@bipt.edu.cn (X.Y.); moyu991018@163.com (Y.M.); gu_yanhong@hotmail.com (Y.G.); 2 Beijing Academy of Safety Engineering and Technology, Beijing 102617, China

**Keywords:** AM60B-Mg alloy, micro-arc oxidation, composite coating, corrosion mechanism, LEIS

## Abstract

In order to improve the corrosion resistance of the automotive AM60B-Mg alloy, a nano-SiO_2_/MAO composite coating was prepared on the surface of the alloy. The electrochemical properties were studied in an 80 °C corrosion environment using potentiodynamic polarization tests. Local Electrochemical Impedance Spectroscopy (LEIS) was used to study the corrosion mechanisms of coating defect zone. The microstructure and phase of the samples were observed by confocal laser microscopy, scanning electron microscopy (SEM) and X-ray diffraction (XRD). Macroscopic electrochemical test results showed that the impedance of the nano-SiO_2_/MAO coating was much higher than that of the MAO coating, by about 433 times. Local electrochemical test results showed that the minimum impedance of the nano-SiO_2_/MAO coating was 1–2 orders of magnitude higher than the maximum impedance of the MAO coating. The defective SiO_2_/MAO coating still had high corrosion resistance compared to the defective MAO coating. A physical model of local corrosion mechanisms was proposed.

## 1. Introduction

Magnesium alloys are preferred to many other known light metals for the manufacture of sheet metal components commonly used in the automotive industry because of their high strain hardening rates and plastic strain ratios [1,2,3]. The density of magnesium is around 33% lower than that of aluminum and approximately a fifth of that of steel. Therefore, there is an opportunity to save fuel by using this in vehicle components, and, consequently, to achieve the required CO_2_ emission reductions [4]. Nevertheless, it is also important to evaluate the mechanical properties resulting from the utilisation of magnesium alloys in car parts and to ensure that they can meet the technical requirements of the automotive sector [5]. Magnesium alloys have strong reactivity, poor corrosion resistance and poor high temperature resistance. In response to possible corrosion, several methods for preventing corrosion on Mg alloys have been used—one of which is coating. Coating is an economical method of erosion protection that can prevent contact between the Mg substrate and corrosive medium. Numerous coating technologies have been introduced for Mg and its alloys for corrosion protection, including chemical conversion coating, micro-arc oxidation (MAO), plasma electrolytic oxidation (PEO), organic coating, cold spraying and layered double hydroxide (LDH) [6].

The MAO process is often used in order to obtain a hard and thick coating with strong adhesion and to protect magnesium alloys from corrosion [7]. However, the MAO coating on the Mg alloy contains pores that can introduce corrosion ions directly into the Mg substrate and initiate corrosion [8]. Therefore, additives are added to the anodizing technology to improve the barrier properties of the coating. A common electrolyte used in the MAO process for magnesium alloys is an alkaline solution containing additives such as aluminum [9,10,11], nanoparticles, etc. The corrosion resistance of Mg alloys can be improved by adding nanoparticles such as SiC, Ag, and Al_2_O_3_ to the MAO coating. The incorporation of certain nanoparticles into MAO coatings to modify and improve the coating structure by closing pores and defects has proven to be easy to manipulate and effective [12,13]. Nano-SiO_2_ has low costs, excellent wear resistance and chemical inertia, and is also often used as an additive for MAO and other coatings. For example, when nano-SiO_2_ is added to a MAO coating on the surface of a 7A52 Aluminum alloy, experimental results show that it can improve the wear resistance and corrosion resistance of the MAO coating [14]. At present, there are relatively few studies on the effects of nano-SiO_2_ on the corrosion resistance of magnesium alloy MAO coatings. In studies on the corrosion properties of MAO coatings, many researchers have used global electrochemical testing methods to give the “average response” of samples, while few people have studied the corrosion properties of MAO coatings after local damage by local electrochemical impedance spectroscopy (LEIS). In fact, LEIS suggests that the corrosion process in the defect zone develops predominantly at the magnesium/coating interface, as a pit-like nano-pore system will grow on the surface after MAO treatment [15,16,17,18].

In this paper, the effects of nano-SiO_2_ on the corrosion resistance of a MAO coating was studied by comparing the MAO coating with or without nano-SiO_2_. A 3.5% NaCl solution was used to simulate the corrosion of chloride ions by melting salt. The solution temperature was 80 °C, which is about the highest temperature reached by auto parts. The coatings before and after corrosion were characterized using a metallographic microscope, laser confocal microscope, scanning electron microscope (SEM) and X-ray diffractometer (XRD). The corrosion resistance of two samples (SiO_2_/MAO coating, MAO coating) were studied by global electrochemical tests, including open circuit potential, electrochemical impedance spectroscopy and potentiodynamic polarization. The local corrosion resistance of coatings with artificial scratches was studied by a LEIS test. The local corrosion mechanism of coating scratches was plotted. Although the MAO process is known to improve the corrosion aspects of Al and Mg-type valve metals, fatigue is equally important for real life conditions in aerospace and automotive parts [19,20]. In a further study, the fatigue properties of the coating will be discussed.

## 2. Materials and Methods

### 2.1. Materials and MAO Process

The chemical composition of the AM60B-Mg alloy material used in this experiment is shown in Table 1. The size of the samples used for the MAO treatment was 25 mm × 25 mm × 5 mm. Before the MAO process, the samples were ground with different grades of sandpaper, such as 400, 600 and 1000, and polished with diamond abrasive paste. Then, the samples were washed with acetone, ethanol and deionized water for 2 min, and immediately dried with cold air.

The equipment used in the MAO process was manufactured by Haoning ™ Electronic Technology Co., LTD from Xi ’an, China. (Model HNMAO-20). The electrolyte used for the MAO treatment was a basic silicate solution, whose composition is shown in Table 2. During the micro-arc oxidation process, the voltage increased linearly from 0–200 V in 150 s, from 200–500 V in 5 min, and finally remained at 500 V for 8 min. The sample was then cleaned with deionized water and blow-dried. The prepared coatings were divided into MAO and SiO_2_/MAO.

### 2.2. Surface Characteristics of the MAO Coating

A LEICA S9D camera was used to take macroscopic images of the sample surfaces. The microstructures were observed by confocal laser microscopy (KH-8700, HIROX from Shanghai, China) and scanning electron microscopy (SEM, FEI Quante 650F from Brook, Germany). An X-ray diffractometer (D8-Focus, BRUKER from Brook, Germany) was used to analyze the surface material composition of the samples before and after corrosion. The Cu target wavelength was 1.5406 angstrom or 0.15406 nm. The scanning range was 10–90°, and the scanning speed was 4°/min.

### 2.3. Corrosion Electrochemical Tests

The MAO-treated samples were cut into blocks of 10 mm × 10 mm × 3 mm. One of the 10 mm × 10 mm surfaces was polished until the alloy substrate was exposed, and then the copper wire was attached to it with conductive adhesive. Finally, the samples were placed in epoxy resin, exposing only the surfaces of the 10 mm × 10 mm coating without being connected to the copper wire, and the encapsulated samples were used for electrochemical testing. A NaCl solution with a mass fraction of 3.5% was used as the corrosion solution and test electrolyte.

#### 2.3.1. Global Electrochemical Tests

Global electrochemical measurements were performed in a 3.5 wt.% NaCl solution at 80 °C using a conventional three-electrode cell: a platinum sheet was used as a counter electrode and silver wire as the reference electrode. The sample was covered with Teflon, except for the 10 mm × 10 mm working electrode. Except for the 10 mm × 10 mm exposed electrode, the samples were covered with Teflon. After immersing the sample in the corrosive solution for 8 h, electrochemical tests were carried out with a workstation (AMETEK, VersaSTAT-3F, Princeton, NJ, USA). The data acquisition time setting for the open circuit potential was 1200 s. The scanning frequency of the EIS was 10^−2^ to 10^5^ Hz and the excitation voltage was 10 mV. The dynamic potential polarization test was carried out with a voltage sweep range of −250 to 500 mV (relative to the open circuit potential), and the sweep rate was 0.5 mV/s.

#### 2.3.2. Local Electrochemical Tests

Local electrochemical tests were measured by a VersaSCAN™ (Princeton, NJ, USA) electrochemical scanning system. An artificial scratch was made on the surface of the coating to amplify the defect effect (the scratch was approx. 1.5 mm long and 0.2 mm wide). A four-electrode system was used for the measurements, in which the sample was used as the working electrode, the saturated glycury electrode (SCE) was the reference electrode, the platinum electrode was the counter electrode and the fourth electrode was a special probe (P/N-224114). The scanning probe working frequency was 1000 Hz, the scanning area was 2 mm × 2 mm, the excitation voltage is 10 mV, the X and Y direction step was 100 μm/s and the probe movement speed and measurement speed were 1000 μm/s and 100 μm/s, respectively.

## 3. Results and Discussion

### 3.1. Characteristics of MAO Coatings

**Figure 1:**
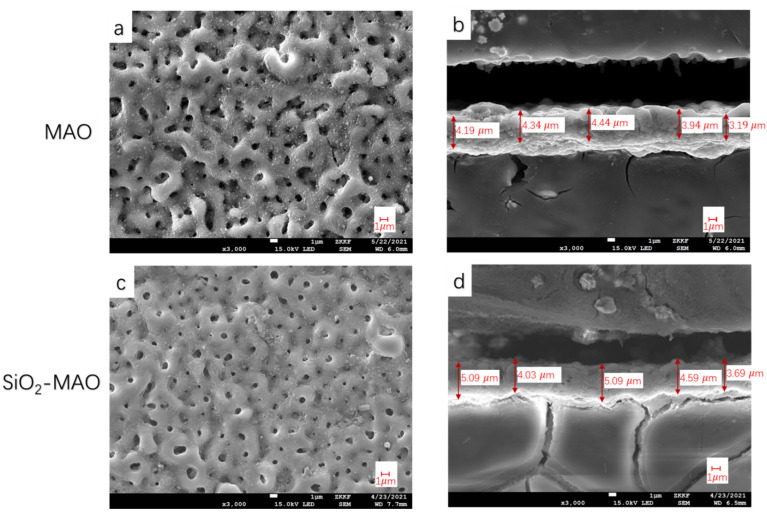
Micromorphology of coatings, (**a**,**b**) Surface and cross-section morphology of the MAO coating, (**c**,**d**) Surface and cross-section morphology of the SiO_2_/MAO coating.

Figure 1 shows the microscopic morphology of two kinds of micro-arc oxide films tested by SEM. Figure 1a,b shows the surface and cross section micromorphology of the MAO coating; Figure 1c,d shows the surface and cross section micromorphology of the SiO_2_/MAO coating. Both coatings had micropores because of the different cooling rates of the molten oxide and the escape of gases during the micro-arc oxidation reaction. The microporosity of the nano-SiO_2_/MAO coating was smaller and less than that of the MAO coating. The cross-section morphology of the two samples is shown in Figure 1b,d. It can be seen that compared to the MAO coating, the nano-SiO_2_/MAO coating was denser and uniform. In the process of the coating preparation, the micro-arc plasma discharge produced instantaneous high temperatures and high pressure, which resulted in chemical oxidation, electrochemical oxidation and plasma oxidation on the surface of the magnesium alloy substrate [21]; the coating grew directly on the substrate, thus bonding very tightly with the substrate. It also can be seen from the figure that the SiO_2_/MAO coating (4.03–5.09 μm) was thicker than the MAO coating (3.19–4.44 μm), indicating that the addition of inorganic nanomaterials can make the coating thicker.

**Figure 2:**
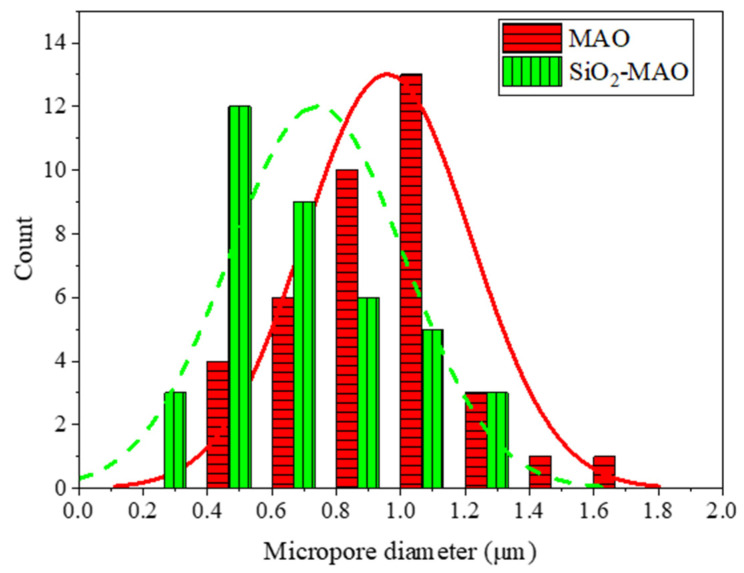
Distribution diagram of micropore diameter on the surface of the MAO coating and SiO_2_/MAO coating.

As can be seen in Figure 2, the microporous sizes of the MAO coating ranged from 0.5 to 1.8 μm, with most of them being between 0.85 and 0.9 μm and 1.05 to 1.1 μm, and the micropore size of the nano-SiO_2_/MAO composite coating was 0.5 to 0.55 μm and 0.7 to 0.75 μm. The reason for this is that nanoparticles were embedded into the MAO coating by electrophoresis and diffusion [22]. Smaller micropores mean that less corrosive material penetrates the channels of the coating. At the same time, the density of the coating was also improved by inorganic nano additives.

**Figure 3:**
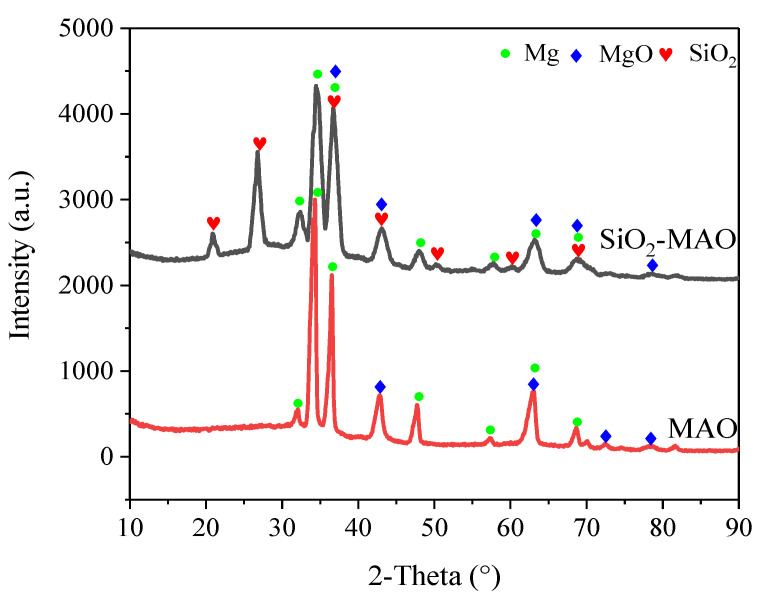
XRD scan results of the MAO coating and SiO_2_/MAO coating.

Figure 3 shows the X-ray diffraction detection results of the MAO coating and SiO_2_/MAO coating. The relatively strongest peaks of the two coatings were Mg and MgO, respectively. The X-rays penetrated the coating and reached the alloy matrix; thus, Mg appeared in the substance peaks. MgO was the main component of the micro-arc oxidation coating. The presence of SiO_2_ peaks indicated that the nanoparticles had been integrated into the coating. The formation mechanism of MgO is similar to that of conventional anodic oxidation; MgO was formed at the membrane/electrolyte and substrate/membrane interfaces.

The EDS compositions of the two coatings are shown in Table 3, which shows that the atomic percentage of O elements was 52.3% and that of Si elements was 7.8%, proving the existence of SiO_2_ particles in the composite coating. Due to the large amount of O elements still present in the electrolyte system, the content ratio of Si elements to O elements was less than 1:2.

### 3.2. Analysis of Global Electrochemical Test Results

#### 3.2.1. Open Circuit Potential (OCP)

**Figure 4:**
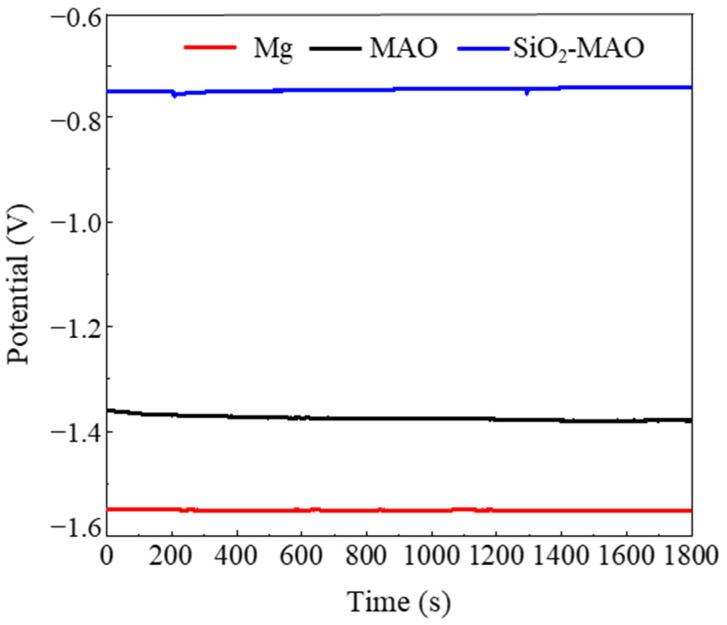
The OCP curves of the AM60B-Mg, MAO coating and SiO_2_/MAO coating.

Figure 4 shows the OCP curves of the three samples soaked at 80 °C for 8 h. With the prolongation of continuous determination time, the OCP value of the samples tended to be stable. The OCP test results showed that the SiO_2_/MAO composite coating had the largest positive potential, followed by the MAO coating, and that the magnesium alloy substrate sample had the largest negative potential—indicating that the SiO_2_/MAO composite coating had the highest corrosion resistance trend.

#### 3.2.2. Electrochemical Impedance Spectroscopy (EIS)

**Figure 5:**
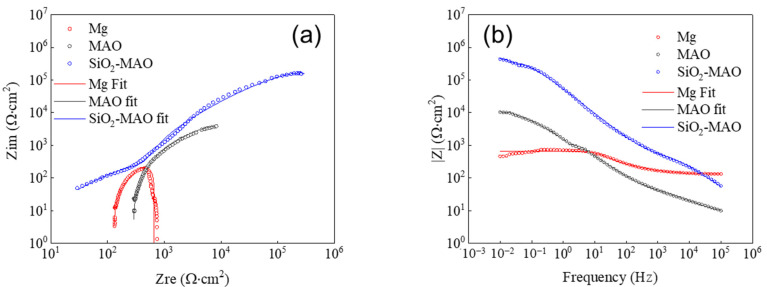
The electrochemical impedance spectroscopy (**a**) Nyquist plot, (**b**) Bode plot.

The electrochemical impedance spectrum of the coating is shown in Figure 5. The circle represents the measured data, and the solid line represents the fitting data obtained by the fitting circuit.

The Nyquist curve in Figure 5a could be approximated as a circular arc, and the larger the radius of the arc, the better corrosion resistance of the sample had. The radius of the corrosion resistance arc from large to small was SiO_2_/MAO coating > MAO coating > magnesium alloy substrate.

The |Z| value at 0.01 Hz in Figure 5b is the modal value of the impedance for the test piece; the higher |Z| value is, the greater corrosion resistance the sample has. The |Z| values of different samples are listed in Table 4, having the same regularity as the Nyquist curve. The |Z| value of the SiO_2_/MAO coating was 185.5 KΩ·cm^2^ > the MAO coating (18.3 KΩ·cm^2^) > the magnesium alloy substrate (0.54 KΩ·cm^2^). The addition of SiO_2_ showed the strongest corrosion resistance and strengthening effect, indicating that the SiO_2_/MAO coating had a good protective effect on the substrate at high temperatures.

**Figure 6:**
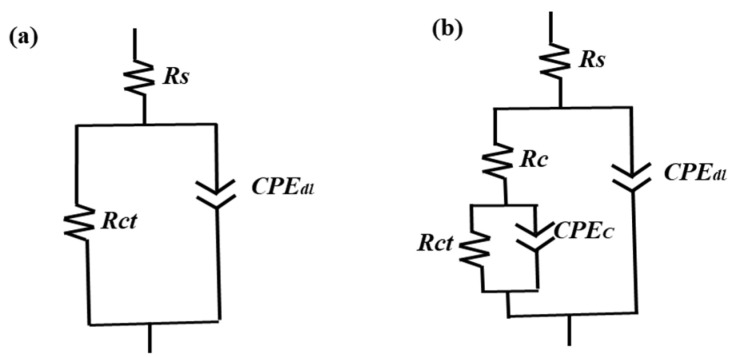
The equivalent circuit model of the (**a**) Mg alloy sample, (**b**) SiO_2_/MAO and MAO coating samples.

The EIS test results were fitted by the equivalent circuit model shown in Figure 6. *R_s_* represents the solution resistance from the sample surface to the reference electrode; *CPE_dl_* and *R_ct_* are the charge transfer resistance and double layer capacitance of the Mg alloy. The values of the parameters represented by the different components in the equivalent circuit diagram are given in Table 5. The *R_ct_* value of the nano-SiO_2_/MAO sample was higher than that of the MAO sample, indicating that the addition of nano-SiO_2_ improves the corrosion resistance of the MAO coating. The reason why pure capacitance C is replaced by *CPE_dl_* is that in the actual electrochemical reaction process, the electrode is affected by surface roughness, porosity and other factors, making the part representing pure capacitance show deviations, which makes it difficult to give reasonable fitting results. Therefore, a constant phase angle element *CPE_dl_* was proposed for fitting.

#### 3.2.3. Potentiodynamic Polarization Scans (PDP)

**Figure 7:**
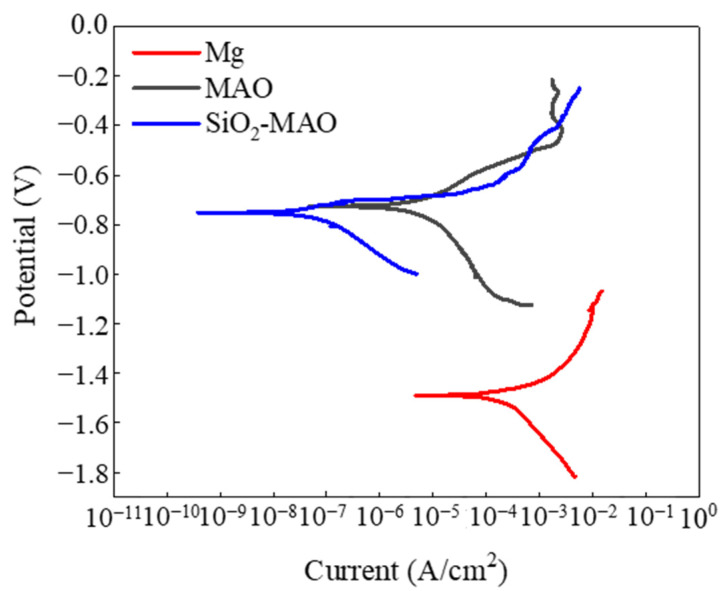
PDP curve of the samples.

The PDP curves of the samples are shown in Figure 7, and the corrosion potentials and current densities of the samples are listed in Table 5. The test results showed that the SiO_2_/MAO coating had the highest positive corrosion potential (−0.73 V) and the lowest corrosion current density (2.45 nA·cm^−2^); the AM60B-Mg alloy substrate had the largest negative corrosion potential (−1.49 V) and the highest corrosion current density (66,200 nA·cm^−2^). The results show that the corrosion resistance of the SiO_2_/MAO coating was much better than the MAO coating and the AM60B-Mg alloy. The PDP curve and EIS result had the same regularity.

#### 3.2.4. Coating Morphology and Material Composition after Corrosion

**Figure 8:**
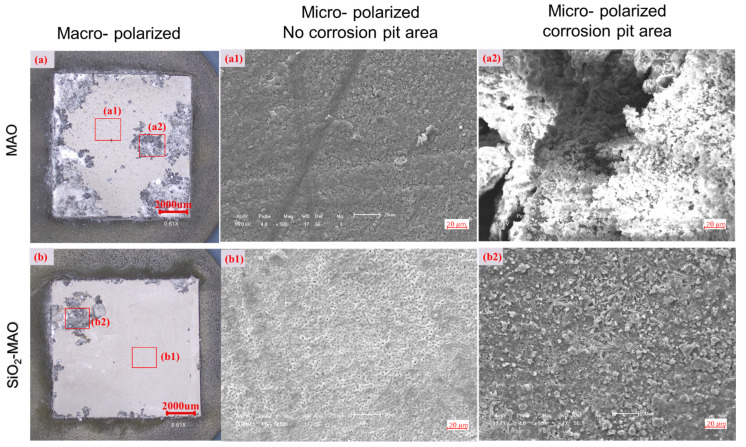
Macroscopic and microscopic appearance of the samples after corrosion. (**a**,**b**) the macroscopic morphology of the MAO coating and SiO_2_/MAO coating after corrosion; (**a1**,**b1**) microscopic morphology of the corrosion-free pit area of the two coatings; (**a2**,**b2**) the microscopic morphology of the corrosion pit area of the two coatings.

Figure 8a,b show the comparison of the macroscopic morphology of the two coating samples after corrosion. It can be seen from the two figures that the corrosion started from the perimeter and expanded to the center, and that the corrosion mode was pitting corrosion. The pitting pits of the MAO coating were much more extensive than that of the SiO_2_/MAO coating. Figure 8(a1,b1) show the microscopic morphology of the corrosion-free pit area of the two coatings; both kinds of coatings remained relatively smooth, but the SiO_2_/MAO coating appeared to have smaller micropore morphology. Figure 8(a2,b2) show the microscopic morphology of the corrosion pit area of the two coatings—the MAO coating cracked and collapsed downward, while the SiO_2_/MAO coating remained flat, and the acicular material formed on the surface was the corrosion product Mg(OH)^2^ [23]. The SEM results following corrosion prove that the addition of nano-SiO_2_ enhanced the corrosion resistance of the MAO coating.

**Figure 9:**
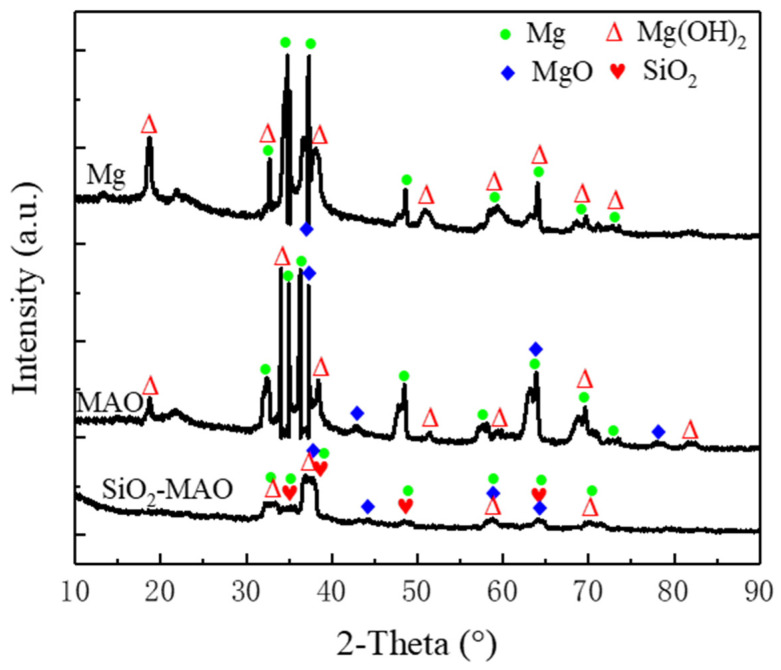
XRD results of the material phase of each sample after corrosion.

Figure 9 shows the XRD patterns of the three samples etched at 80 °C for 8 h. The results showed that there was a common corrosion product Mg(OH)_2_ on the surface of the three samples. In addition to the co-existing Mg(OH)_2_ phase, the Mg phase was also present on the surface of the magnesium alloy; there were Mg and MgO phases on the MAO coating surface, and SiO_2_, Mg and MgO phases could be detected on the nano-SiO_2_/MAO-coated surface. Compared with the magnesium alloy substrate, the number of Mg(OH)_2_ peaks in the MAO coating decreased by 1, indicating that the MAO process can slow down the corrosion rate of the magnesium alloy. The Mg(OH)_2_ peak number of the SiO_2_/MAO coating was the lowest, indicating that the nano-SiO_2_ improved the corrosion resistance of the MAO coating. The main reaction equations for the coating in the corrosive solution are as follows [24,25]:

Mg − 2e^−^ = Mg^2+^ (1)

2H_2_O + 2e^−^ = H_2_ + 2OH^−^ (2)

2Mg^2+^ + 2OH − 2e^−^ = 2MgO + H_2_ (3)

MgO = Mg^2+^ + O^2−^ (4)

Mg^2+^ + 2OH^−^ = Mg(OH)_2_ (5)

### 3.3. Analysis of Local Electrochemical Test Results

MAO coatings can protect the AM60B-Mg alloy substrate, but the coating on the surface of the alloy is inevitably damaged due to external mechanical forces. Corrosion failure usually begins at the site of the coating damage, and corrosion pits may be the source of fatigue cracks. Due to the potential difference between the coating and the magnesium alloy substrate, once the damaged coating is in contact with a corrosive medium, galvanic corrosion will occur, affecting the service life and safety of the AM60B-Mg alloy. The corrosion behavior of coatings with defects was studied by means of local electrochemical tests.

**Figure 10:**
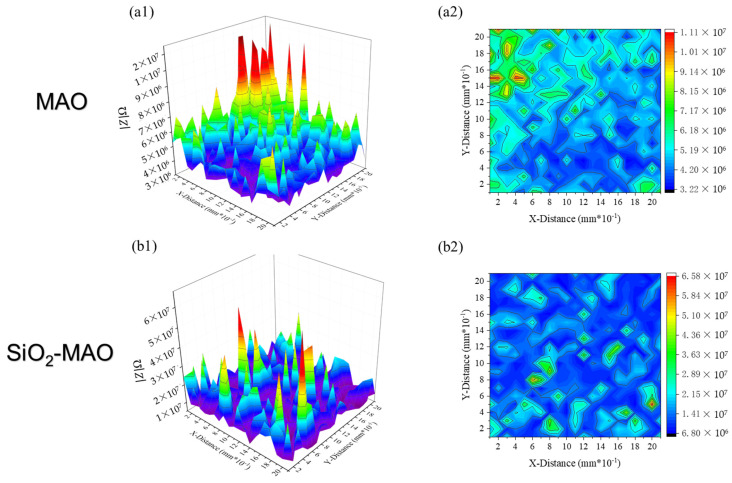
Local impedance of the non-scratched coating, (**a1**,**a2**) MAO coating, (**b1**,**b2**) SiO_2_/MAO coating.

Figure 10 shows the local impedance distribution of the non-scratched coating (scanning area was 2 mm × 2 mm). It can be seen from the figure that the resistance value of the SiO_2_/MAO coating (6.80 × 10^6^~6.58 × 10^7^ Ω) was significantly higher than that of the MAO coating (3.22 × 10^6^~1.11 × 10^7^ Ω), indicating that the SiO_2_/MAO coating had better corrosion resistance.

**Figure 11:**
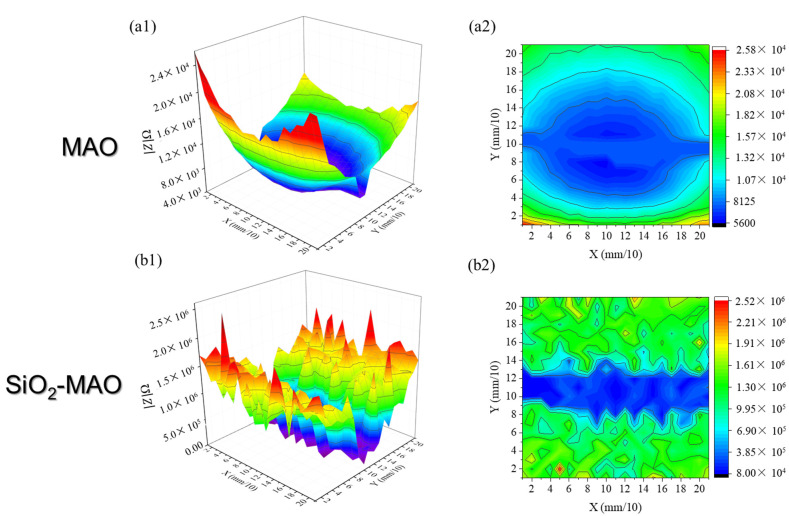
Local impedance with scratched coatings: (**a1**,**a2**) MAO coating, (**b1**,**b2**) SiO_2_/MAO coating.

Figure 11 shows the local impedance results for two coatings with scratches (the scan area was 2 mm × 3 mm and the scratch was located in the center of the scan area). The impedance at the scratch was the lowest, and gradually increased from the scratch to both sides. A regular “V-shaped valley” was formed after the MAO scratches were damaged, while an irregular “V-shaped valley” was formed after the SiO_2_/MAO scratches, indicating that the original appearance of the micropores (less corrosion) was still maintained near the scratches. In the case of coating defects, the impedance of the MAO coating was 5.60 × 10^3^~2.58 × 10^4^ Ω, and the impedance of the SiO_2_/MAO coating was 8.00 × 10^4^~2.52 × 10^6^ Ω; the SiO_2_/MAO coating still showed high corrosion resistance.

**Figure 12:**
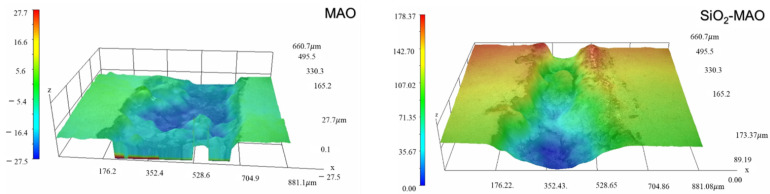
Local microscopic appearance of coating scratches.

Figure 12 is a local micrograph of coating scratches observed using a confocal laser microscope. It can be seen that serious electrochemical corrosion occurred at the edge of the scratch, which is the boundary between the substrate and the coating. The MAO coatings showed severe corrosion at the scratch, while the SiO_2_/MAO coatings showed slightly less corrosion than the MAO coatings, and there was no particularly large corrosion area.

### 3.4. Corrosion Process Model of Coatings

**Figure 13:**
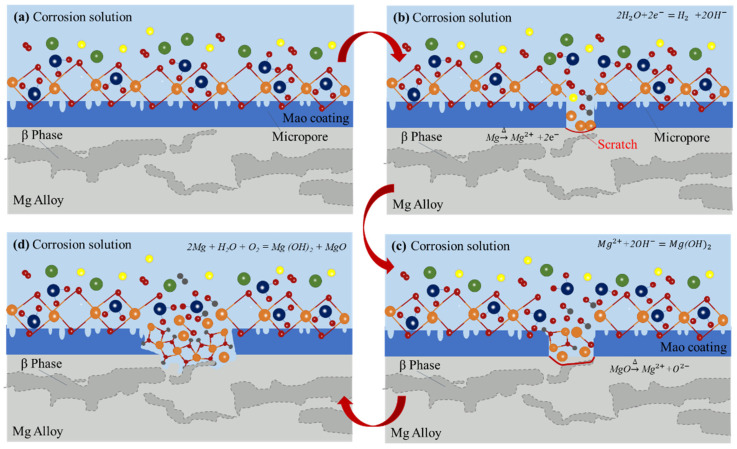
Local corrosion process model of coatings. (**a**) The sample coated with a complete coating is placed in a corrosive solution; (**b**) The coating is scratched by uncontrollable factors such as external mechanical forces; (**c**) The magnesium alloy substrate begins to oxidize when contacting with the corrosion solution, and the coating loses its support, leading to cracking; (**d**) The local corrosion at the coating defect gradually expands to form corrosion pits.

The local corrosion process model was built as shown in Figure 13. Figure 13a shows a situation where a complete coating sample was placed in a corrosive solution and the coating completely sealed the substrate in contact with the corrosive solution. The coating was affected by external mechanical forces and other uncontrollable factors, resulting in scratches on the coating, as shown in Figure 13b. After the coating was destroyed, the matrix came into contact with the corrosion solution, and the magnesium alloy matrix began a slow oxidation reaction. The reaction equations were as follows [23]:

2Mg + H_2_O + O_2_ = Mg(OH)_2_ + MgO (6)

Mg^2+^ + 2H_2_O = Mg(OH)_2_ + H_2_ (7)

In this process, magnesium absorbs oxygen and corrodes, forming Mg(OH)_2_ and MgO (Equation (6)). At the same time, some of the magnesium ions enter the corrosion solution and hydrolyze; Mg(OH)_2_ is also produced (Equation (7)), and corrosion products begin to accumulate in the scratch area. After a period of corrosion, the substrate under the scratch is corroded and dissolved, resulting in the formation of a cavity. The coating on the top of the cavity then loses the support of the original matrix and is subjected to various external forces, eventually resulting in cracking, as shown in Figure 13c. There are an α phase and β phase in the magnesium alloy matrix. When the coating was defective, the corrosion development at the scratch location was determined by whether it was in the α-phase or β-phase. When corrosion occurs in the α phase, the electrode potential of the α phase is negatively correlated with the potential of the oxide film, and the α phase corrodes as an anode; the corrosion process will continue into the Mg alloy matrix. When corrosion occurs in the β-phase, the electrode potential of the β-phase is positively correlated with the potential of the oxide film, and the β-phase is not corroded, while the nano-SiO_2_/MAO film is corroded as an anode [26]. As the corrosion time continues to increase, the local corrosion at the coating defect will follow the pattern shown in Figure 13, gradually expanding the corrosion and eventually forming a corrosion pit, as shown in Figure 13d.

## 4. Conclusions

The corrosion resistance of MAO coatings with or without nano-SiO_2_ particles on an AM60B-Mg alloy was studied in this work. Through surface characterization and electrochemical analysis, the following conclusions were drawn:

(1) The microstructure of the coating showed that the SiO_2_/MAO composite coating had fewer micropores and higher flatness. The thickness of the SiO_2_/MAO coating was larger than the thickness of the MAO coating. As a sealant, nano-SiO_2_ was able to reduce the number of micropores in the MAO coating and thicken the coating. Mg(OH)_2_ corrosion products were found in both coatings after corrosion, and the corrosion mode was pitting corrosion, without large area corrosion.
(2) The global electrochemical test results showed that the SiO_2_/MAO coating had stronger corrosion resistance. The corrosion current density of the MAO coating was 400 times higher than that of the SiO_2_/MAO coating. The results of local electrochemical tests in the low impedance region where the scratches were located showed that the impedance value of the MAO coating was one order of magnitude lower than that of the SiO2/MAO coating. The longitudinal corrosion depth and transverse corrosion width of the SiO_2_/MAO coating were both lower than those of the MAO coating. The SiO_2_/MAO coatings had a much lower corrosion tendency, even in the case of defective coatings.
(3) A model for the local corrosion process of the SiO_2_/MAO coating was constructed. When the coating was scratched, the corrosion development of the defect location depended on whether the corrosion point was in the α phase or the β phase. The alloy substrate was corroded and dissolved first, and then the coating lost the support of the substrate; cracks and spalling appeared under the action of external forces and finally led to the appearance of corrosion pits.
(4) The study results can expand the application of magnesium alloys in aerospace and automotive lightweight metal research directions.

## Figures and Tables

**Table 1:** The chemical composition of the AM60B-Mg alloy (wt.%).

Chemical Composition	Si	Mn	Cu	Fe	Zn	Al	Ni	Mg
wt.%	0.025	0.27	0.0017	0.0017	0.05	5.9	0.0014	Bal.

**Table 2:** Table showing the solution composition (g(mL)/L).

Na_2_SiO_3_	Na_3_PO_4_	KOH	KF	C_3_H_8_O_3_	Nano-SiO_2_ Powder
5 g/L	5 g/L	2 g/L	5 g/L	2 mL/L	5 g/L

**Table 3:** Weight percentage and atomic percentage of the surface elements of two coatings.

Sample	Element	At%
MAO	O	53.7
	Mg	46.3
SiO_2_/MAO	O	52.3
	Mg	39.9
	Si	7.8

**Table 4:** Fitting impedance parameters of each sample after soaking at 80 °C.

	R_s_ (Ω·cm^2^)	R_c_ (Ω·cm^2^)	CPEc (Ω^−1^·cm^−2^·s-n)	CPE_dl_ (Ω^−1^·cm^−2^·s-n)	R_ct_ (Ω·cm^2^)
Mg	13.7			0.720	537
MAO	15	84	0.5463	0.6731	18257
SiO_2_/MAO	6.647	2409	0.8851	0.9113	185,500

**Table 5:** Potentiodynamic polarization curve fitting results.

Sample	Mg	MAO	SiO_2_MAO
**E_corr_/V**	−1.49	−0.77	−0.73
**I_corr_/nA·cm^2^**	66200	1120	2.45

## Data Availability

All data needed to evaluate the conclusions in the paper are present in the paper. Additional data related to this paper may be requested from the authors.
